# Examining Secular Changes in Health Risk Behavior Profiles and Their Associations With Mental Distress During Adolescence

**DOI:** 10.3389/ijph.2026.1609345

**Published:** 2026-04-07

**Authors:** Laura Bechtiger, Hans Thalathara, David Bürgin, Lukas Eggenberger, Clarissa Janousch

**Affiliations:** 1 Interdisciplinary Center for Psychopathology and Emotion Regulation, Department of Psychiatry, University Medical Center Groningen, Groningen, Netherlands; 2 Jacobs Center for Productive Youth Development, University of Zurich, Zurich, Switzerland; 3 Child and Adolescent Psychiatric Research Department, University Psychiatric Hospitals, University of Basel, Basel, Switzerland; 4 Experimental and Clinical Pharmacopsychology, Department of Adult Psychiatry and Psychotherapy, University Hospital of Psychiatry Zurich, University of Zurich, Zurich, Switzerland; 5 Karolinska Institute, Department of Global Public Health, Stockholm, Sweden

**Keywords:** adolescence, health behavior in school-aged children study, health-risk behavior, latent profile analysis, mental distress, substance use, secular change

## Abstract

**Objectives:**

Adolescent mental distress has increased in recent decades. It is unclear whether this is associated with changes in health-risk behaviors.

**Methods:**

We analyzed five waves (2002–2018) of the repeated cross-sectional Swiss Health Behavior in School-aged Children study (ages 11–15; *N* = 30,122). Latent Profile Analyses identified health-risk behavior profiles in each wave using five indicators (physical inactivity, poor sleep, unhealthy diet, smoking, alcohol use). Associations with sociodemographic variables and mental distress (internalizing and somatic symptoms, life satisfaction) were tested using multinomial and linear regressions, including profile*sex interactions.

**Results:**

A consistent four-profile solution (low-risk; high alcohol use/slightly elevated substance use; moderate substance use; highest risk) fit best across waves. The low-risk profile was most prevalent and increased in later cohorts (2014–2018), while elevated-risk profiles declined. Older adolescents were more likely to belong to elevated-risk groups, which were associated with greater mental distress, especially in earlier cohorts. No significant sex interactions were found.

**Conclusion:**

Health-risk behavior profiles remained stable, but their associations with mental distress weakened over time. Prevention efforts should adapt to evolving adolescent contexts.

## Introduction

Recent years have seen the rise of a youth mental health crisis. Although this issue has gained particular attention during and in the aftermath of the COVID-19 pandemic [1, 2], mental distress in young people has already been increasing prior to the pandemic [3]. This mental health crisis is characterized by higher levels of internalizing symptoms, somatic complaints, and reduced life satisfaction in more recent cohorts of adolescents, especially among girls [3–7]. Globally, approximately one in five children and adolescents experience depression or elevated depressive symptoms, underscoring the substantial burden of youth mental distress [8]. Research on potential drivers of the youth mental health crisis examined manifold factors, ranging from the rise of social media to sexual violence exposure to macro-economic factors, and typically yielded mixed findings for many indicators (see 8,9 for reviews). Some important and malleable risk factors have received less research attention as explanatory factors for secular changes in mental distress, including health-risk behaviors. Yet, understanding such malleable risk factors is of special interest for public health, considering the potential to foster youth mental health at the population level [9].

Why would health-risk behaviors be among the potential explanatory variables? First, health-risk behaviors, such as physical inactivity, an unhealthy diet, poor sleep, and legal or illegal substance use, have repeatedly been associated with mental distress [10–14]. Second, health-risk behaviors are also known to differ between males and females [15, 16]: During adolescence, females tend to report more physical inactivity than males [17], whereas males tend to report more unhealthy eating habits and more substance use than females [18–20]. Third, both gender differences in the prevalence of mental distress and gender differences in health-risk behavior have been found to emerge in or by early adolescence [21–24]. This makes the early to mid-adolescent period an important developmental time to understand this association.

Some evidence suggests that, next to secular trends in mental distress, health-risk behaviors show secular change as well. The direction of this change depends on the specific health behaviors in question. A recent overview notes that alcohol and tobacco use have declined over the past two decades, while other risks remain persistent concerns, reflecting changing patterns of adolescent health [25]. Consistent with this broader picture, the direction of change varies by behavior: physical activity and fitness have declined [26, 27], and adolescents now appear to sleep for shorter durations than earlier cohorts [28, 29], whereas smoking and drinking have decreased in many settings [30–32].

Not only has the prevalence of mental distress and some health-risk behavior changed in recent decades, but there is additional evidence that the associations between mental distress and health-risk have also changed across that timespan. For instance, the association between substance use and mental distress has grown stronger in adolescents born in the early 2000s compared to adolescents born in the early 1990s, despite the decrease in the overall prevalence of substance use [33]. This stronger association between substance use and mental distress was particularly observed in females [33]. In contrast, another study has found that mental distress and behavioral risk are less strongly associated in more recent than earlier cohorts of adolescents [34].

Existing studies on secular changes in health-risk behavior and the associations with mental distress have focused on specific behaviors rather than examining changes in patterns. Because risk and protective behaviors frequently cluster, analyzing single behaviors or simply adjusting one for another may obscure shared determinants [35]. Examining health-risk behavior profiles instead of isolated behaviors offers a more comprehensive view of risk [36, 37], as the risk for mental distress increases with the accumulation and co-occurrence of risk behaviors, potentially in a non-linear manner [11]. Two broad strategies can be used to define behavioral risk patterns: theory-driven classification based on predefined thresholds, and empirical classification using mixture models [36, 38]. While theory-based approaches offer conceptual clarity, they require validated and developmentally invariant cut-offs across behaviors that differ substantially in scale, distribution, and social meaning. In adolescent populations, such thresholds are often unavailable or vary across age and historical context. Empirical mixture models, therefore, provide a pragmatic approach for summarizing co-occurring behaviors without imposing *a priori* subgroup boundaries. Therefore, secular changes in health behavior profiles over the past two decades need to be systematically examined on their own and in relation to changes in mental distress, given preliminary evidence that the association between mental distress and health-related risk could have changed in recent decades [7].

### The Current Study

This study leverages publicly available data from the Swiss Health Behavior in School-Aged Children study (HBSC), a repeated cross-sectional survey conducted from 2002 to 2018. Using five time points, we examined secular changes in health-risk behavior profiles (i.e., physical inactivity, poor diet, poor sleep, and substance use) and potential changes in the association between health-risk behaviors and mental distress across this period among adolescents aged 11, 13, and 15 years. In contrast to prior research that has focused solely on individual health-risk behaviors, we attempted to identify co-occurring behavioral risk patterns using Latent Profile Analysis (LPA). We also investigated whether the associations between the resulting health-risk profiles and three distinct, yet complementary indicators of adolescent mental health and wellbeing, namely, internalizing symptoms, somatic symptoms, and life satisfaction, changed across time. Given that recent increases in adolescent mental health problems have been particularly pronounced among girls, we further examined gender differences in both profile prevalence and associations with mental distress.

## Methods

### Recruitment and Participants

The HBSC study is an international comparative survey conducted in collaboration with the World Health Organization (WHO). This repeated cross-sectional study collects data every 4 years to monitor health behaviors, outcomes, and contextual factors among adolescents across >40 countries [39, 40]. This study uses publicly available data from the Swiss HBSC study (2002–2018), which is part of the international HBSC research network [41, 42]. The Swiss HBSC surveys adhered to a standardized international protocol to ensure consistency and comparability across countries and time periods.

Participants were recruited through a two-stage cluster sampling design. In the first stage, schools were randomly selected to represent various geographical regions of Switzerland. In the second stage, entire classes within these schools were randomly chosen to participate. This approach ensured a nationally representative sample of the target population, adolescents aged 11, 13, and 15 years (i.e., 5th, 7th, and 9th grades). For the current study, a total of 30,122 participants (50.3% girls) were included. Data were collected using standardized, self-administered paper-and-pencil questionnaires completed during school hours under teacher supervision. Responses were anonymized.

The study adhered to strict ethical guidelines as outlined in the HBSC international protocol. Parental consent was obtained where required, and participants were informed about the voluntary nature of their participation. Ethical approval was granted by the relevant Swiss authorities for each survey cycle.

### Measures

#### Risky Health Behavior

Physical inactivity was measured using the question: Over the past 7 days, on how many days were you physically active for a total of at least 60 min per day? Responses ranged from 0 days (0) to 7 days [7]. Responses were reverse-coded so that higher scores indicate lower physical activity.

Poor sleep: Participants were asked about their sleep difficulties over the last 6 months with the question: “How often have you experienced difficulties falling asleep?” Response options ranged from about every day [1] to rarely or never [5]. Responses were reverse-coded so higher scores indicate poorer sleep.

Poor diet: Participants were asked four items about their dietary habits: “How many times a week do you usually eat fruits/eat vegetables/eat sweets/drink Coke or other soft drinks?” Items were rated on a seven-point scale, ranging from never [1] to more than once daily [7]. Responses for fruit and vegetable consumption were reverse-coded so that higher scores indicated lower consumption. A mean value was calculated across these four variables.

Smoking: From 2002 to 2014, smoking was assessed with the item “How often do you smoke tobacco at present?” and rated on a four-point scale from every day [1] to never [4]. In 2018, smoking was assessed with the item “In the past 30 days, on how many days (if any) have you smoked cigarettes?”, which was rated on a seven-point scale from never [1] to 30 days (or more) [7]. In 2014, when both items were included to assess smoking behavior, the 2018 item was used in those instances where the earlier smoking variable was not available (97/6634 cases). In order to harmonize the variables across the waves, we reversed-coded the item values from 2002 to 2014 so that higher values indicated more frequent smoking. We rescaled the 2018 variable to align with the 1-4 range while retaining the 7-point coding.

Alcohol use: From 2002 to 2014, alcohol use was assessed with three items “At present, how often do you drink anything alcoholic, such as beer/such as wine/such as spirits?” and rated on a five-point scale from every day [1] to never [5]. In 2018, alcohol use was assessed with the item “In the past 30 days, on how many days (if any) have you drunk alcohol?”, which was rated on a seven-point scale from never [1] to 30 days (or more) [7]. Again, to harmonize the variables across the waves, we used the maximum frequency reported for beer/wine/spirits for 2002 to 2014, respectively, before reverse-coding the mean scores so that higher values indicated more frequent alcohol use. Also, we rescaled the 2018 variable to align with the 1-5 range while retaining the 7-point coding.

#### Health Outcome Variables

Internalizing symptoms: Participants rated the frequency of feeling nervous, dizzy, low, and irritable on a five-point Likert scale, ranging from about every day [1] to rarely or never [5]. A reverse mean score was generated, with higher scores indicating more internalizing symptoms. Cronbach’s alphas are shown in the Supplementary Table S1.

Somatic symptoms: Participants rated the frequency of headaches, stomachaches, and backaches within the last 6 months on a five-point Likert scale, ranging from about every day [1] to rarely or never [5]. A reverse mean score was generated, with higher scores indicating more frequent somatic symptoms. Cronbach’s alphas are shown in the Supplementary Table S1.

Life satisfaction: Respondents indicated their perceived life satisfaction on a 10-point Likert scale, ranging from the worst possible life (0) to the best possible life (10) [43].

All of these measures are intended to capture population-level variation in mental distress and wellbeing rather than to assess clinical disorders, and have shown to have adequate validity and reliability in previous research [44–46].

#### Sociodemographic Variables

Sociodemographic characteristics included in the analysis were age (i.e., ages 11, 13, or 15), self-reported participant gender (1 = male; 2 = female), and family socioeconomic status (SES). As an indicator of family SES, a version of the Family Affluence Scale [47] was coded as the sum of four items that were included at each assessment (i.e., having a family car, target participant having their own bedroom, the number of computers in the house, and number of family holidays abroad in the past year).

### Statistical Analysis

LPA was conducted using Mplus (Version 8.10; [48]), R version 4.4.2 [49] and the MplusAutomation package [50] to identify distinct subgroups of health behaviors separately for each time point but across all age groups. The analysis included five health-risk indicators: physical inactivity, sleep problems, unhealthy diet, smoking, and alcohol consumption. Variables were z-standardized to account for measurement variability. Models were estimated with the maximum likelihood with robust standard errors (MLR) estimator. Variance within profiles was set to zero to facilitate model estimation. Full information maximum likelihood (FIML [51])was used to account for missing data.

To determine the optimal number of latent classes, models ranging from one to six classes were evaluated, and their fit was compared using several statistical criteria, including the Akaike Information Criterion (AIC; [52]), the Bayesian Information Criterion (BIC; [53], sample size-adjusted BIC (aBIC; [53, 54]), entropy values, the Lo-Mendell-Rubin Likelihood Ratio Test (LMR-LRT; [55]), the Vuong-Lo-Mendell-Rubin Likelihood Ratio Test (VLMR-LRT; [55]), and the Bootstrapped Likelihood Ratio Test (BLRT; [56–58]. Final model selection balanced parsimony with interpretability, guided by substantive relevance and statistical fit indices (see Supplementary Material for an overview of model indices; Supplementary Table S2).

To explore the association between distinct risky behavior profiles and sociodemographic characteristics and health outcomes, we saved the most likely profile membership from the LPA models and used it as an outcome/predictor in subsequent regression models. We estimated four sets of regression models: First, we examined the association between sociodemographic variables and health-risk behavior profiles using multinomial logistic regression. Second, we examined health risk behavior profiles as bivariate predictors (dummy-coded) of mental distress, using linear regression models. Third, we examined health-risk behavior profiles as predictors of wellbeing, adjusting for sociodemographic characteristics. Fourth, we also included the profile*sex interactions in the adjusted models. As a sensitivity analysis, we additionally estimated health-risk profiles across all waves combined and examined their associations with sociodemographic characteristics and mental distress.

## Results

Table 1 presents descriptive statistics of the main study variables per survey wave (see also Supplementary Figures S1, S2 for age-specific and sex-specific trend graphs).

**TABLE 1:** Descriptive statistics of main study variables. Health Behavior in School Aged Children study, Switzerland, 2002–2018.

Variables	2002 (*N* = 4,679)			2006 (*N* = 4,621)			2010 (*N* = 6,678)			2014 (*N* = 6,634)			2018 (*N* = 7,510)		
	*n*	*M*	*SD*	*n*	*M*	*SD*	*n*	*M*	*SD*	*n*	*M*	*SD*	*n*	*M*	*SD*
Health risk behaviors															
Alcohol use	4,586	1.75	1.04	4,579	1.59	0.93	6,595	1.70	0.99	6,579	1.47	0.83	7,412	1.19	0.49
Smoking	4,661	1.34	0.86	4,603	1.21	0.67	6,636	1.25	0.73	6,579	1.15	0.56	7,463	1.09	0.43
Physical inactivity	4,633	3.11	2.01	4,517	3.25	1.96	6,615	3.03	1.81	6,525	2.84	1.86	7,438	2.84	1.87
Sleep problems	4,655	1.17	1.23	4,501	1.33	1.31	6,549	1.33	1.33	6,511	1.44	1.34	7,439	1.40	1.37
Unhealthy diet	4,673	3.33	1.01	4,595	3.13	1.00	6,667	3.18	1.00	6,614	3.11	0.98	7,506	3.03	0.98
Mental distress															
Internalizing symptoms	4,663	2.01	0.78	4,512	2.10	0.83	6,563	2.04	0.83	6,526	2.12	0.84	7,551	2.09	0.86
Life satisfaction	4,646	7.82	1.71	4,464	7.78	1.80	6,537	7.70	1.84	6,448	7.73	1.76	7,405	7.69	1.85
Somatic symptoms	4,667	1.85	0.79	4,519	1.90	0.83	6,576	1.92	0.86	6,529	1.97	0.84	7,552	1.94	0.85

### Latent Profile Analysis

**FIGURE 1:**
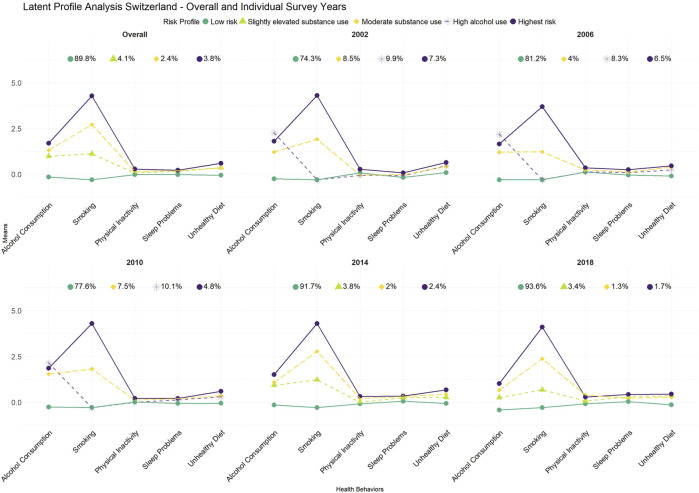
Health-risk behavior profiles for each assessment. Health Behavior in School Aged Children study, Switzerland, 2002–2018.

The LPA analysis suggested that a four-profile solution best fit the data at all time points. The resulting health-risk behavior profiles are illustrated in Figure 1. When estimating profiles separately within each wave, the most frequent health-risk behavior profile with a prevalence of 74.3%–93.6% was always a low-risk profile, characterized by low values on each risk behavior. Second, we identified high alcohol use (2002–2010) and slightly elevated substance use (2014–2018) profiles, which showed elevated rates of substance use compared to the low-risk profile, though at varying degrees depending on the time point (prevalence: 3.4%–10.1%). Third, we identified a moderate substance use profile that was characterized by high levels of substance use (prevalence: 1.3%–8.5%). Fourth, we identified a highest risk profile, with elevated rates of substance use but also slightly elevated risk for other health-risk behaviors (prevalence: 1.7%–7.3%). Thus, profiles mainly differed in their substance use behavior (Figure 1; Supplementary Figures S1, S2).

A secular trend in the prevalence of health risk behaviors was observed. More adolescents were assigned to a low-risk group in 2014 and 2018 compared to the earlier years. This is reflected in a decrease in the prevalence of all three other groups in 2014 and 2018 compared to the previous years.

### Sociodemographic Predictors of Latent Profile Membership

Table 2 shows sociodemographic predictors of profile membership across the years. Most consistently, older adolescents were more likely to be in the high alcohol use/slightly elevated substance use, moderate substance use, or highest risk profile than younger adolescents. Age also discriminated between these three health-risk profiles. Relative to 11-year-olds, for 13- and 15-year-olds the odds rose for highest risk vs. high alcohol use/slightly elevated substance use and for moderate substance use vs. high alcohol use/slightly elevated substance use. Sex differences are primarily seen in the years from 2002 to 2010. Compared with boys, girls had lower odds of belonging to any elevated-risk profile vs. low-risk. However, conditional on elevated risk, girls were more likely to be in moderate substance use vs. high alcohol use/slightly elevated substance use and more likely to be in highest risk vs. high alcohol use/slightly elevated substance use in 2002.

**TABLE 2:** Associations of sociodemographic characteristics with health-risk behavior profiles. Health Behavior in School Aged Children study, Switzerland, 2002–2018.

Sociodemographic variables	High alcohol use/slightly elevated substance use vs. low-risk		Moderate substance use vs. low-risk		Highest risk vs. low-risk		Moderate substance use vs. high alcohol use/slightly elevated substance use		Highest risk vs. high alcohol use/slightly elevated substance use		Moderate substance use vs. highest risk	
	OR	95% CI	OR	95% CI	OR	95% CI	OR	95% CI	OR	95% CI	OR	95% CI
2002												
Sex	**0.40**	0.32–0.49	0.85	0.68–1.06	**0.65**	0.51–0.83	**2.14**	1.62–2.82	**1.63**	1.22–2.18	1.31	0.97–1.77
SES	**1.13**	1.02–1.25	0.99	0.89–1.11	0.92	0.81–1.04	0.88	0.77–1.01	**0.81**	0.70–0.94	1.08	0.93–1.26
Age	**2.93**	2.60–3.31	**2.52**	2.22–2.86	**4.99**	4.16–5.98	0.86	0.73–1.01	**1.70**	1.39–2.09	**0.50**	0.41–0.62
2006												
Sex	**0.42**	0.33–0.53	**0.61**	0.45–0.82	0.82	0.64–1.05	**1.46**	1.01–2.09	**1.96**	1.43–2.69	1.35	0.93–1.96
SES	**1.15**	1.03–1.29	0.89	0.77–1.04	0.90	0.79–1.02	**0.78**	0.65–0.93	**0.78**	0.67–0.91	1.00	0.83–1.20
Age	**2.82**	2.46–3.23	**2.24**	1.89–2.66	**4.33**	3.60–5.22	**0.79**	0.64–0.98	**1.54**	1.23–1.91	**1.93**	1.51–2.47
2010												
Sex	**0.56**	0.47–0.66	**0.60**	0.50–0.73	**0.64**	0.51–0.82	1.09	0.86–1.38	1.16	0.88–1.52	0.94	0.70–1.25
SES	**1.19**	1.09–1.30	1.09	0.99–1.20	0.93	0.83–1.05	0.91	0.81–1.03	**0.78**	0.68–0.89	**1.17**	1.01–1.34
Age	**2.68**	2.42–2.97	**2.82**	2.51–3.18	**5.38**	4.42–6.55	1.05	0.91–1.23	**2.01**	1.62–2.48	**0.52**	0.42–0.65
2014												
Sex	**0.73**	0.56–0.95	0.81	0.57–1.15	0.82	0.59–1.14	1.11	0.72–1.70	1.12	0.74–1.68	0.99	0.62–1.58
SES	**1.22**	1.06–1.41	0.99	0.83–1.19	0.93	0.78–1.10	0.81	0.65–1.01	**0.76**	0.61–0.94	1.07	0.84–1.36
Age	**1.93**	1.67–2.23	**2.64**	2.11–3.31	**5.45**	4.04–7.35	**1.37**	1.05–1.79	**2.82**	2.03–3.92	**0.49**	0.33–0.70
2018												
Sex	0.98	0.75–1.27	0.86	0.57–1.29	**0.63**	0.43–0.92	0.88	0.54–1.41	0.64	0.41–1.01	1.36	0.79–2.35
SES	1.09	0.95–1.25	1.07	0.87–1.31	0.88	0.74–1.04	0.98	0.77–1.25	**0.81**	0.65–0.99	1.21	0.93–1.58
Age	**2.93**	2.46–3.48	**6.27**	4.22–9.33	**5.65**	4.02–7.93	**2.14**	1.40–3.29	**1.93**	1.32–2.81	1.11	0.66–1.86

OR, odds ratio; CI=confidence interval. Coefficients are bolded when the confidence interval does not cross zero.

### Mental Health Outcomes of Health-Risk Behavior Profile Membership

Table 3 shows the results from bivariate and multivariate regression analyses of health-risk behavior profiles predicting outcomes. Bivariately, assignment to the high alcohol use/slightly elevated substance use profile was associated with more somatic and internalizing symptoms in 2010 compared to those assigned to the low-risk profile. Assignment to the moderate substance use profile was associated with increased somatic symptoms in 2002 and 2010, with more internalizing symptoms in 2002 and 2010, and with lower life satisfaction in 2010 compared to those assigned to the low-risk profile. Assignment to the highest risk profile was associated with increased somatic symptoms in 2002 and 2010, more internalizing symptoms in 2002 and 2006, and lower life satisfaction in 2002, 2006, and 2010 compared to those assigned to the low-risk profile. Moreover, highest risk profiles were significantly associated with more somatic symptoms, more internalizing symptoms, and lower life satisfaction. Except for the difference between the highest risk profile compared to the low-risk profile in somatic symptoms and life satisfaction in 2010, these findings held up when accounting for socioeconomic covariates. Between the three elevated risk profiles, differences in somatic symptoms, internalizing symptoms, and life satisfaction were small and inconsistent across waves.

**TABLE 3:** Regression results of the health-risk behavior profiles-mental distress association. Health Behavior in School Aged Children study, Switzerland, 2002–2018.

Regression models	Bivariate						Multivariate					
	Somatic symptoms		Internalizing		Life satisfaction		Somatic symptoms		Internalizing		Life satisfaction	
	β	95% CI	β	95% CI	β	95% CI	β	95% CI	β	95% CI	β	95% CI
Reference category: low-risk												
2002												
High alcohol use/slightly elevated substance use	0.05***	−0.04, 0.14	0.07***	−0.02, 0.16	−0.04**	−0.14, 0.05	0.07***	−0.03, 0.16	0.09***	−0.01, 0.18	−0.06***	−0.15, 0.04
Moderate substance use	0.11***	0.01, 0.21	0.12***	0.02, 0.23	−0.10***	−0.20, 0.01	0.11***	0.00, 0.21	0.12***	0.02, 0.23	−0.09***	−0.20, 0.02
Highest risk	0.13***	0.02, 0.24	0.17***	0.06, 0.28	−0.15***	−0.26, −0.04	0.13***	0.02, 0.25	0.18***	0.06, 0.29	−0.14***	−0.25, −0.02
2006												
High alcohol use/slightly elevated substance use	0.07***	−0.03, 0.18	0.06***	−0.05, 0.16	−0.08***	−0.18, 0.03	0.08***	−0.03, 0.19	0.06***	−0.05, 0.17	−0.07***	−0.18, 0.04
Moderate substance use	0.06***	−0.08, 0.21	0.08***	−0.06, 0.23	−0.12***	−0.27, 0.03	0.06***	−0.08, 0.21	0.08***	−0.01, 0.23	−0.11***	−0.25, 0.04
Highest risk	0.11***	−0.00, 0.23	0.13***	0.02, 0.25	−0.17***	−0.29, −0.05	0.10***	−0.02, 0.22	0.13***	0.00, 0.25	−0.14***	−0.27, −0.02
2010												
High alcohol use/slightly elevated substance use	0.10***	0.02, 0.20	0.08***	0.00, 0.16	−0.05***	−0.13, 0.03	0.10***	0.02, 0.18	0.09***	0.01, 0.17	−0.04**	−0.12, 0.04
Moderate substance use	0.11***	0.01, 0.23	0.11***	0.02, 0.21	−0.12***	−0.22, −0.03	0.11***	0.01, 0.20	0.12***	0.03, 0.21	−0.11***	−0.20, −0.02
Highest risk	0.12***	0.02, 0.18	0.10***	−0.01, 0.22	−0.14***	−0.25, −0.02	0.11***	−0.01, 0.22	0.11***	−0.01, 0.22	−0.11***	−0.22, 0.01
2014												
High alcohol use/slightly elevated substance use	0.07***	−0.06, 0.19	0.09***	−0.03, 0.22	−0.06***	−0.19, 0.07	0.06***	−0.06, 0.19	0.10***	−0.03, 0.22	−0.06***	−0.18, 0.07
Moderate substance use	0.07***	−0.11, 0.24	0.07***	−0.10, 0.24	−0.10***	−0.27, 0.07	0.06***	−0.11, 0.23	0.07***	−0.10, 0.24	−0.09***	−0.26, 0.08
Highest risk	0.11***	−0.05, 0.26	0.08***	−0.08, 0.24	−0.08***	−0.24, 0.08	0.09***	−0.07, 0.25	0.08***	−0.08, 0.24	−0.06***	−0.23, 0.10
2018												
High alcohol use/slightly elevated substance use	0.08***	−0.05, 0.20	0.11***	−0.02, 0.23	−0.09***	−0.21, 0.04	0.06***	−0.06, 0.19	0.10***	−0.03, 0.22	−0.07***	−0.19, 0.06
Moderate substance use	0.04**	−0.16, 0.24	0.06***	−0.14, 0.26	−0.05***	−0.25, 0.15	0.02*	−0.17, 0.22	0.05***	−0.14, 0.25	−0.04**	−0.24, 0.16
Highest risk	0.10***	−0.08, 0.28	0.09***	−0.09, 0.26	−0.09***	−0.27, 0.09	0.09***	−0.09, 0.27	0.08***	−0.09, 0.26	−0.08***	−0.26, 0.10
Reference category: high alcohol use/slightly elevated substance use												
2002												
Moderate substance use	0.06***	−0.07, 0.19	0.06***	−0.06, 0.19	−0.06**	−0.19, 0.07	0.04*	−0.08, 0.17	0.05*	−0.08, 0.17	−0.04*	−0.17, 0.09
Highest risk	0.09***	−0.04, 0.23	0.12***	−0.02, 0.25	−0.11***	−0.25, 0.02	0.08***	−0.06, 0.21	0.11***	−0.03, 0.24	−0.09***	−0.23, 0.04
2006	​	​	​	​	​	​	​	​	​	​	​	​
Moderate substance use	0.01	−0.16, 0.19	0.04*	−0.13, 0.22	−0.06***	−0.24, 0.11	0.01	−0.16, 0.18	0.04*	−0.13, 0.21	−0.06***	−0.23, 0.11
Highest risk	0.05**	−0.10, 0.20	0.09***	−0.07, 0.24	−0.10***	−0.26, 0.05	0.03	−0.12, 0.18	0.07***	−0.08, 0.22	−0.08***	−0.23, 0.07
2010												
Moderate substance use	0.03	−0.09, 0.14	0.04**	−0.07, 0.16	−0.08***	−0.20, 0.03	0.02	−0.09, 0.13	0.04**	−0.07, 0.16	−0.07***	−0.19, 0.04
Highest risk	0.05***	−0.08, 0.18	0.05**	−0.08, 0.18	−0.10***	−0.23, 0.03	0.04**	−0.09, 0.17	0.04**	−0.09, 0.17	−0.08***	−0.21, 0.05
2014												
Moderate substance use	0.02	−0.19, 0.22	0.00	−0.21, 0.21	−0.05***	−0.27, 0.16	0.04**	−0.15, 0.24	0.00	−0.21, 0.21	−0.05**	−0.26, 0.16
Highest risk	0.05***	−0.15, 0.25	0.01	−0.19, 0.20	−0.03*	−0.23, 0.17	0.01	−0.20, 0.22	0.00	−0.20, 0.20	−0.02	−0.22, 0.18
2018												
Moderate substance use	−0.01	−0.24, 0.22	−0.01	−0.24, 0.22	0.00	−0.23, 0.24	−0.02	−0.24, 0.21	−0.01	−0.24, 0.22	0.01	−0.23, 0.24
Highest risk	0.04**	−0.17, 0.26	0.01	−0.21, 0.22	−0.03**	−0.25, 0.18	0.05***	−0.17, 0.26	0.01	−0.20, 0.23	−0.03*	−0.24, 0.18
Reference category: highest risk												
2002												
Moderate substance use	−0.03	−0.18, 0.11	−0.06***	−0.20, 0.08	0.06***	−0.08, 0.21	−0.04	−0.18, 0.10	−0.07**	−0.21, 0.08	0.06**	−0.09, 0.20
2006												
Moderate substance use	−0.03	−0.21, 0.15	−0.02	−0.21, 0.16	0.02	−0.16., 0.20	−0.02	−0.20, 0.16	−0.02	−0.20, 0.16	0.01	−0.17, 0.19
2010												
Moderate substance use	−0.04*	−0.18, 0.10	−0.01	−0.15, 0.13	0.04*	−0.10, 0.18	−0.03	−0.16, 0.11	−0.01	−0.15, 0.13	0.02	−0.11, 0.16
2014												
Moderate substance use	−0.03*	−0.26, 0.20	−0.00	−0.23, 0.23	−0.03	−0.26, 0.21	−0.03	−0.26, 0.20	−0.00	−0.23, 0.23	−0.03	−0.26, 0.20
2018												
Moderate substance use	−0.05***	−0.31, 0.21	−0.02	−0.28, 0.25	0.03*	−0.23, 0.30	−0.06***	−0.32, 0.20	−0.02	−0.28, 0.24	0.03*	−0.23, 0.30

Multivariate models adjusted for sex, age, and family SES. Coefficients are bolded when the confidence interval does not cross zero.

*p < 0.05; **p < 0.01; ***p < 0.001.

### Sex Differences in the Health-Risk Behavior and Mental Distress Association

Sex-stratified associations were small and imprecise, likely due to attenuated profile–distress links in later cohorts, reduced power in sex-specific subsamples, and wave-specific class composition (see Supplementary Tables S3, S4 for multivariate regression models run separately for males and females and the corresponding profile × sex interaction tests). In males, assignment to the highest risk profile compared to the low-risk profile was associated with more internalizing symptoms in 2002. Assignment to the high alcohol use/slightly elevated substance use profile compared to the low-risk profile was associated with more somatic symptoms in 2010. In females, assignment to the highest risk profile compared to the low-risk profile was associated with more internalizing symptoms in 2002. Assignment to the moderate substance use risk profile compared to the low-risk profile was associated with more internalizing symptoms and lower life satisfaction in 2010. However, confidence intervals for the profile-by-sex interactions all included zero, indicating that associations between health-risk behavior profiles and mental distress indicators did not differ significantly by sex.

### Sensitivity Analysis

Results from the sensitivity analysis with the overall profiles are presented in the Supplementary Tables S5, S6, Supplementary Figure S3. Briefly, the overall profiles reflected the profiles identified for 2014 and 2018 better than the profiles identified for 2002, 2006, and 2010 (i.e., finding a slightly elevated substance use profile instead of a high alcohol use profile). Associations with sociodemographic characteristics changed in the earlier waves in that most associations with sex and SES are not significant, whereas the associations with age remained. Also, most of the associations with mental distress were non-significant, with the exception of the highest risk/low risk contrast in 2002.

## Discussion

The present study examined secular changes in health-risk behavior profiles among Swiss adolescents from 2002 to 2018, using five waves of HBSC data, and how these profiles relate to indicators of mental distress and wellbeing (i.e., somatic symptoms, internalizing symptoms, and life satisfaction). Using latent profile analysis, we identified four distinct health-risk profiles for each time point: a large low-risk group (i.e., low-risk), and three smaller groups (i.e., high alcohol use/slightly elevated substance use; moderate substance use; highest risk), characterized by varying levels of substance use and, to a lesser extent, other risk behaviors. Similar patterns have been reported in other HBSC participant countries, with international HBSC reports documenting declining adolescent substance use alongside stable or increasing mental distress in recent cohorts [40]. Comparable trends have also been observed in Norway, where the population-based Young-HUNT study reported concurrent declines in health-risk behaviors and increases in mental health problems over time [59].

### Secular Trends in Health-Risk Profiles

Across the study period, the prevalence of the low-risk profile increased, as the prevalence of all other health-risk behaviors decreased. This decrease was most pronounced for the profiles characterized by different levels of substance use only (i.e., high alcohol use/slightly elevated substance use; moderate substance use). This pattern is consistent with broader secular trends for lower age-specific prevalence of alcohol consumption and smoking among high-income countries like Switzerland [31, 60, 61], as well as with a previous study that documented an increase of low health-risk behaviors in youth [34]. It should be noted that, due to a change in measurement for alcohol and nicotine use in 2018 compared to the earlier assessments, the exact means of substance use in the profile estimation cannot be directly compared, as the variables required harmonization. Nevertheless, substance use behavior, and smoking in particular, differentiated the high-risk profiles, with minor variability in other behaviors such as sleep, diet, and exercise.

### Associations With Mental Distress and Wellbeing

Associations between health-risk behavior profiles and the three indicators of mental distress were small and inconsistent. Although adverse health-risk behavior profiles were associated with more somatic symptoms, more internalizing symptoms, and a lower life satisfaction than those assigned to the low-risk group, this trend was primarily observed in the first waves (2002–2010). After 2014, these correlations decreased or disappeared, suggesting a potential cohort effect. One potential reason for this is that substance use, historically a marker of psychological vulnerability, may have lost its strong link with mental health as its prevalence declined and social meaning shifted [33]. As substance has become less common among adolescents and young adults, fitness-oriented lifestyles have gained importance [62]. Alternatively, the decoupling of risky behaviors and wellbeing in later cohorts might reflect changes in other risk and protective contexts, such as academic pressure, economic stressors, or improved prevention and support structures [43, 63]. Considering that the associations of health-risk profiles and mental distress did not remain significant in a sensitivity analysis where profiles were estimated based across all survey years and that there are overlapping confidence intervals across the waves, secular changes should be interpreted cautiously.

Variation in mental health outcomes did not vary significantly across the different elevated-risk profiles, suggesting that merely the presence of more than one risk behavior (potentially substance use), rather than their pattern, seems to account for the association with distress [11]. Controlling for sex, age, and SES made a minimal difference in the health-risk behavior and mental distress associations, reaffirming that the observed pattern is not the result of demographic confounding. Although the associations between behavior profiles and mental distress were not significant, health-risk behaviors remain public-health relevant, as previous studies show they predict long-term health outcomes [63]. Even small differences in adolescent health may widen over time, contributing to health inequalities in adulthood [9, 64]. Promoting healthy routines during this formative period can therefore yield lasting benefits for adult wellbeing.

### Gender Interactions

Girls in higher-risk profiles reported slightly more mental health problems than boys, aligning with broader evidence of increasing gender disparities in adolescent internalizing symptoms [4, 23]. However, these differences were small and not statistically significant, as confidence intervals included zero, indicating no reliable sex-based variation in the link between health-risk behaviors and mental distress. Gendered social roles, less adaptive coping mechanisms, and increased susceptibility to psychosocial stressors are some possible explanations for the overall higher burden among girls [65]. Despite the fact that boys were marginally more likely to belong to high-risk groups, our results indicate that intervention and prevention should be attentive to the mental health needs of both genders.

### Further Sociodemographic Differences

Although sociodemographic characteristics did not account for the (small) associations between health-risk behavior profiles and mental distress, associations with health-risk behaviors were observed. Across all waves, older adolescents were more likely to be assigned to one of the substance use profiles. This pattern was to be expected given the age-of-onset of substance use in Switzerland, and is consistent with developmental theories highlighting increased exposure and opportunity for risk behavior with age [64, 66, 67]. Socioeconomic differences were less consistent but suggested that lower family wealth increased the likelihood of being part of the highest risk rather than the low-risk profile. This underscores the importance of targeting prevention resources to vulnerable groups. These associations were overall quite stable, indicating that even as overall risk behaviors decline, age, sex, and SES continue to shape adolescents’ health-behavior clustering.

### Contextual Interpretation

The limited variation in activity, diet, and sleep in the health-risk behavior profiles that emerged from the data analysis could reflect structural protection, such as mandatory physical education at school [68, 69] and the wide availability of healthy foods [70]. In contrast, differences in alcohol and nicotine use differentiate the emerging risk profiles, potentially reflecting their normalization and easy access in Switzerland (i.e., the legal purchasing age for beer and wine is 16 years, and only since October 2024, a nationwide minimum purchase age of 18 applies for tobacco products and e-cigarettes, [71]). Although informal enforcement has tightened in recent years, social norms still determine prevalence and perceived risk [72]. Future research would benefit from examining how shifting cultural and regulatory conditions influence the interrelation between adolescent behavior patterns and mental health. In particular, cross-national comparisons are needed to determine whether the Swiss patterns observed in this study reflect broader international trends or country-specific dynamics shaped by policy and cultural context.

### Strengths and Limitations

This study has several strengths, including the use of large, nationally representative data spanning 16 years, harmonized measures of mental distress and health-risk behaviors across five repeated cross-sectional assessments, and a latent profile approach to capture health-risk behavior patterns across five common health-risk behaviors.

Some limitations must be noted. First, the cross-sectional nature of each assessment makes it impossible to disentangle the direction of the health-risk behavior-mental distress associations. These are likely bidirectional [73]. Second, several measurement comparability limitations need to be addressed. The measurement of alcohol use and smoking behavior changed in 2018 compared to the earlier assessments, which may have introduced bias due to reduced comparability. Since the aim of this study was not to compare absolute means but to understand relative differences in alcohol/smoking frequency between groups at the same time point, we consider this harmonization appropriate for our analytic goals. In addition, binge or heavy episodic drinking could not be examined, as this measure was not assessed consistently across survey waves, which may limit the identification of more problematic patterns of alcohol use. Future HBSC waves that include binge drinking indicators could help distinguish frequency of alcohol use from more harmful drinking patterns and clarify their links with adolescent mental distress. Furthermore, e-cigarette use was not assessed prior to 2022, and sleep was limited to difficulties falling asleep, without information on sleep maintenance or sleep duration, which may underestimate certain contemporary risk behaviors and sleep-related problems. Third, mental distress outcomes were assessed using brief self-report scales designed for large-scale surveillance rather than clinical diagnosis. In particular, the somatic symptom scale should be interpreted as an index of symptom burden rather than a unidimensional latent construct, which may partly explain modest internal consistency. Fourth, all data were self-reported, which can be especially problematic in studies of non-conforming behaviors, such as risky behaviors. Indeed, such underreporting has been documented and is driven by factors like social desirability, stigma, and fear of negative consequences [74, 75]. Lastly, latent profile approaches are exploratory by nature and depend on model selection choices [76]. LPA solutions should not be interpreted as discrete or naturally occurring subgroups but may reflect continuous variation in health-risk behavior. Profile definitions are also dependent on model specification and indicator selection and are therefore best understood as descriptive summaries. We decided to estimate wave-specific profiles to align with the repeated cross-sectional design of the HBSC study that allows to compare different snapshots across time. At the same time, theory-driven classifications face comparable limitations, including reliance on arbitrary cut-offs and limited flexibility in the context of secular change.

### Conclusion

In summary, our study shows that adolescent health-risk behavior profiles remain a useful way to capture co-occurring behaviors, but their associations with mental distress are inconsistent and have weakened in more recent cohorts. This shift may reflect changing social meanings of substance use and structural protections in Switzerland. While prevention efforts should continue to target adolescent substance use because of its established health risks, these findings suggest that health-risk behaviors are less likely to be contributing significantly to the ongoing mental health crisis, highlighting the need to reassess which behavioral patterns are most relevant for adolescent mental health today.
