# Supplementary material for: Examining Secular Changes in Health Risk Behavior Profiles and Their Associations With Mental Distress During Adolescence
Source: Int J Public Health. 2026 Apr 7;71:1609345. doi: 10.3389/ijph.2026.1609345 (PMC13096746; doi:10.3389/ijph.2026.1609345)
Supplement: Supplementary file 1 [file Supplementaryfile1.docx]

**Supplement**


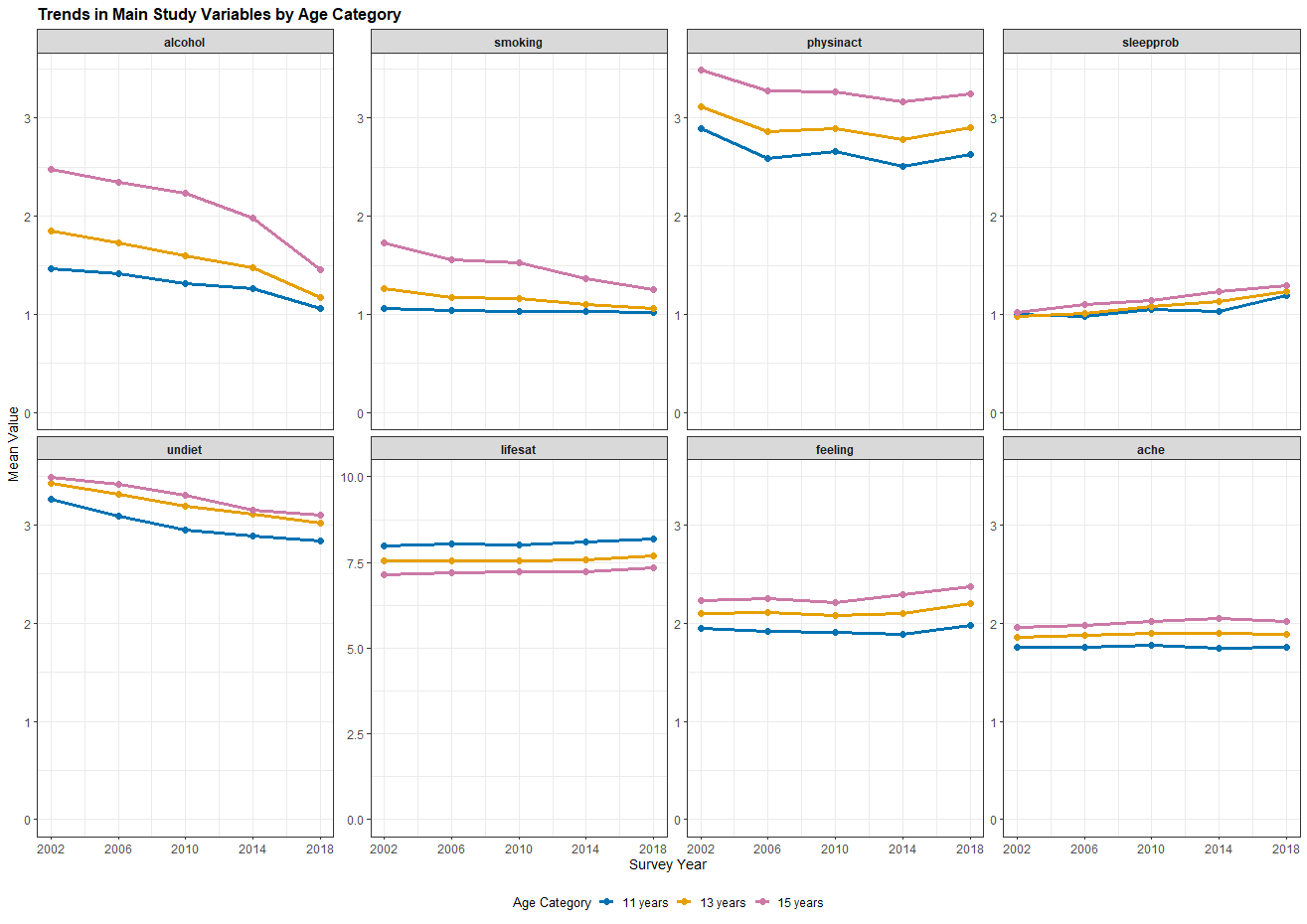


Figure S1. Trends in main study variables by age category. Health Behaviour in School-Aged Children study, Switzerland, 2002-2018.


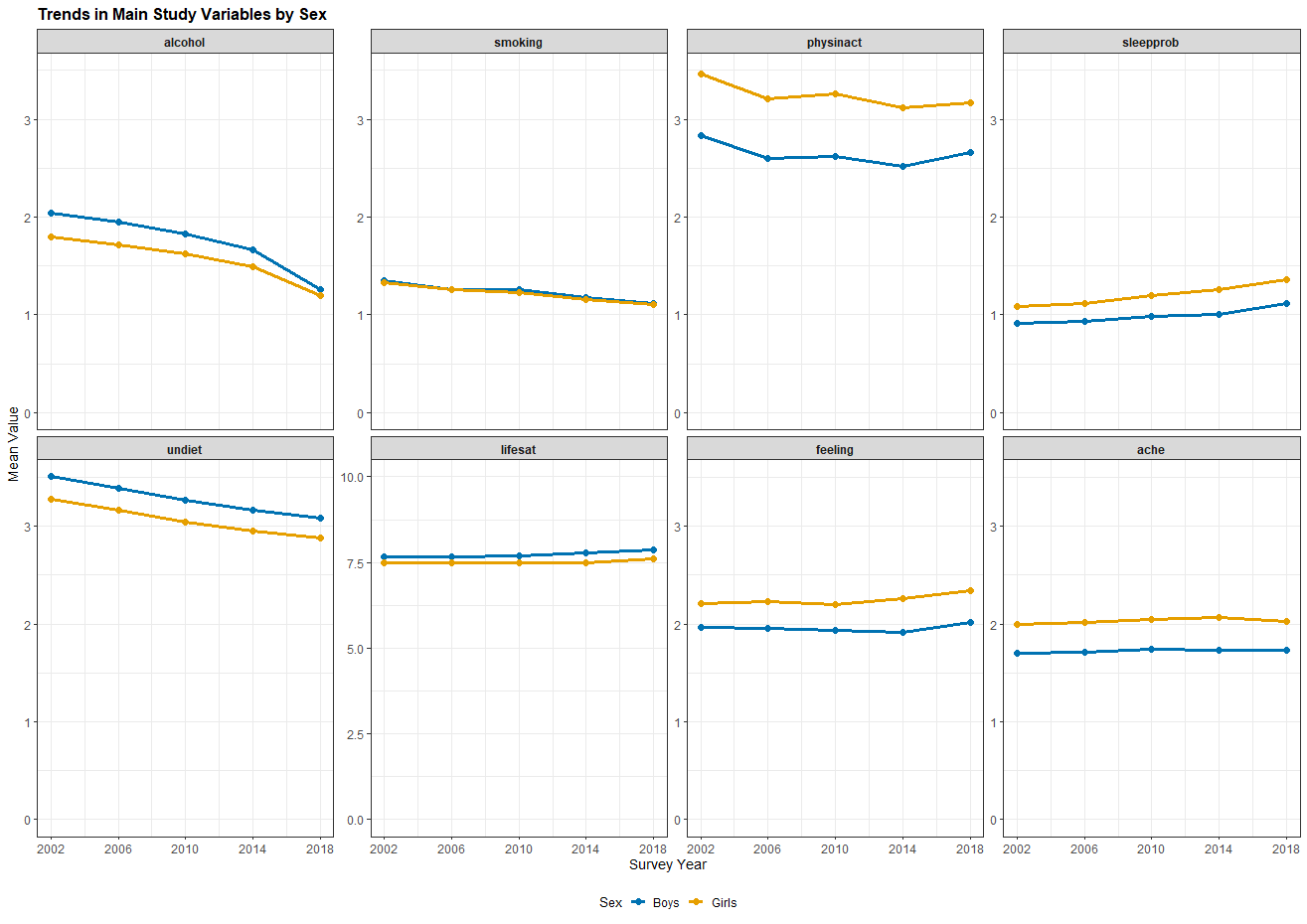


Figure S2. Trends in main study variables by sex. Health Behaviour in School-Aged Children study, Switzerland, 2002-2018.


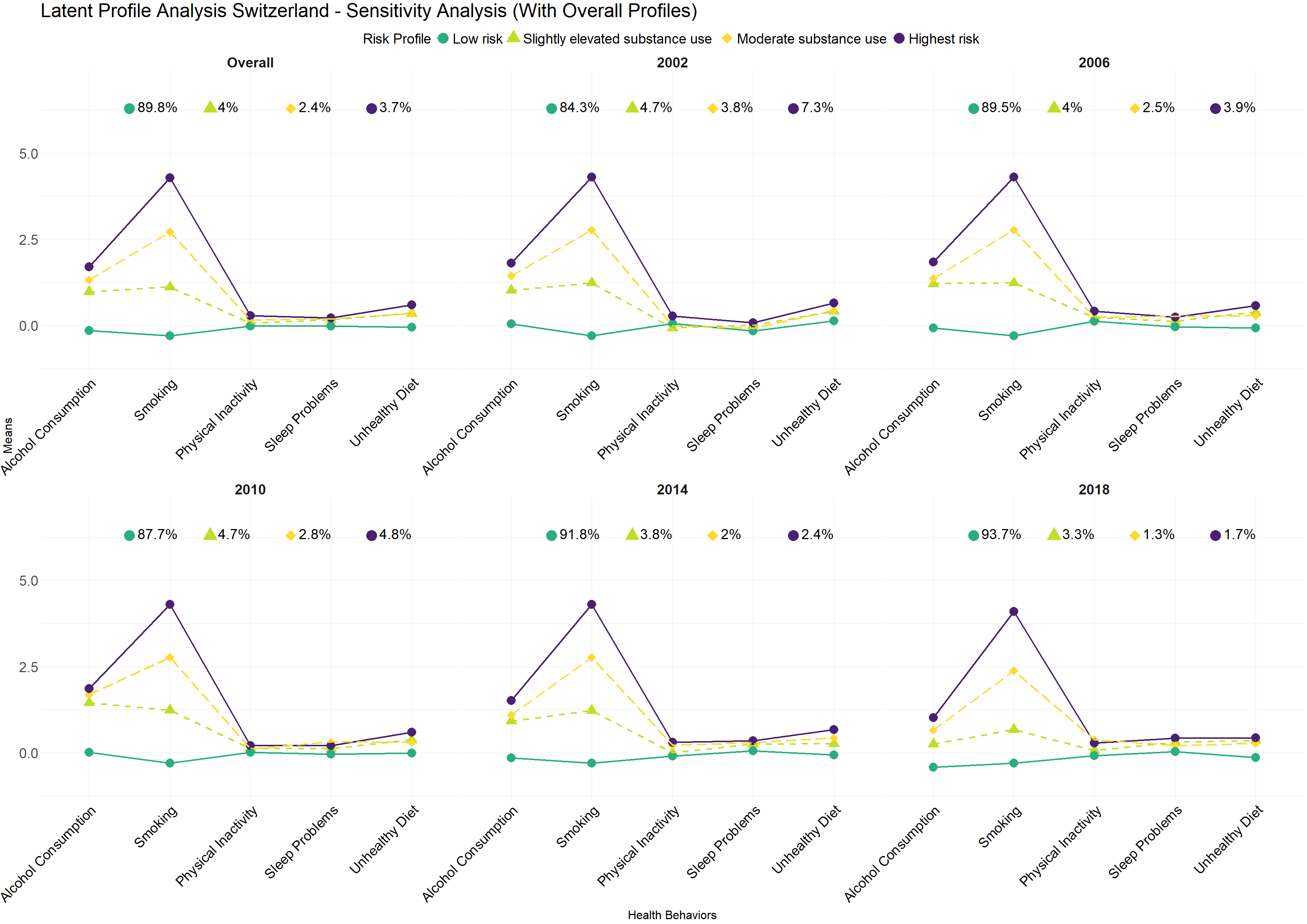


Figure S3. Sensitivity analysis showing the distribution of latent profiles derived from the overall pooled model across survey waves. Health Behaviour in School-Aged Children study, Switzerland, 2002-2018.

Table S1. Cronbach’s alphas of mental distress variables. Health Behaviour in School-Aged Children study, Switzerland, 2002-2018.

|  | 2002 | | 2006 | | 2010 | | 2014 | | 2018 | |
| --- | --- | --- | --- | --- | --- | --- | --- | --- | --- | --- |
| Mental distress | # of items | α | # of items | α | # of items | α | # of items | α | # of items | α |
| Internalizing symptoms | 4 | 0.724 | 4 | 0.741 | 4 | 0.731 | 4 | 0.736 | 4 | 0.728 |
| Life satisfaction | 1 | - | 1 | - | 1 | - | 1 | - | 1 | - |
| Somatic symptoms | 3 | 0.612 | 3 | 0.629 | 3 | 0.658 | 3 | 0.620 | 3 | 0.632 |

*Note*. Single-item for life satisfaction.

Table S2. Summary statistics for model selection. Health Behaviour in School-Aged Children study, Switzerland, 2002-2018.

|  | k | LL | BIC | aBIC | AIC | Entropy | BLRT_PValue | | T11_VLMR_PValue | | T11_LMR_PValue | Observations | ClassificationProbRange | ClassSizes_n_Percent |
| --- | --- | --- | --- | --- | --- | --- | --- | --- | --- | --- | --- | --- | --- | --- |
| Overall |  |  |  |  |  |  |  | |  | |  |  |  |  |
|  | 1 | -211605 | 423313.6 | 423281.8 | 423230.4 |  |  |  | |  | 30122 | 1 - 1 | 100% |  |
|  | 2 | -183720 | 367604.4 | 367553.5 | 367471.3 | 0.998 | 0 | | 0 | | 0 | 30122 | 0.994 - 1 | 93.9%, 6.1% |
|  | 3 | -169296 | 338819.7 | 338749.7 | 338636.8 | 0.998 | 0 | | 0 | | 0 | 30122 | 0.994 - 1 | 3.8%, 5.8%, 90.4% |
|  | 4 | -140544 | 281377.7 | 281288.8 | 281145 | 0.998 | 0 | | 0 | | 0 | 30122 | 0.993 - 1 | 4.1%, 2.4%, 89.8%, 3.8% |
|  | 5 | -124779 | 249908.1 | 249800 | 249625.4 | 0.998 | 0 | | 0.2876 | | 0.2913 | 30122 | 0.993 - 1 | 0.6%, 89.8%, 3.6%, 3.6%, 2.3% |
|  | 6 | -120150 | 240713.3 | 240586.2 | 240380.8 | 0.988 | 0 | | 0 | | 0 | 30122 | 0.966 - 0.997 | 0.6%, 82.8%, 2.4%, 3.4%, 7%, 3.8% |
| 2002 |  |  |  |  |  |  |  | |  | |  |  |  |  |
|  | 1 | -34945.5 | 69975.41 | 69943.64 | 69910.9 |  |  | |  | |  | 4679 | 1 - 1 | 100% |
|  | 2 | -30561.2 | 61257.61 | 61206.76 | 61154.39 | 0.998 | 0 | | 0 | | 0 | 4679 | 0.997 - 1 | 88.9%, 11.1% |
|  | 3 | -28582.8 | 57351.5 | 57281.59 | 57209.58 | 0.998 | 0 | | 0 | | 0 | 4679 | 0.995 - 1 | 8.5%, 84.2%, 7.3% |
|  | 4 | -28127.9 | 56492.34 | 56403.37 | 56311.72 | 0.956 | 0 | | 0 | | 0 | 4679 | 0.946 - 0.996 | 8.5%, 74.3%, 9.9%, 7.3% |
|  | 5 | -27848.3 | 55983.9 | 55875.86 | 55764.57 | 0.972 | 0 | | 0 | | 0 | 4679 | 0.925 - 0.993 | 73.2%, 8.5%, 2.5%, 11%, 4.8% |
|  | 6 | -27705.2 | 55748.42 | 55621.32 | 55490.39 | 0.888 | 0 | | 0 | | 0 | 4679 | 0.666 - 0.993 | 16.2%, 8.5%, 57.3%, 10.7%, 4.8%, 2.5% |
| 2006 |  |  |  |  |  |  |  | |  | |  |  |  |  |
|  | 1 | -32852.9 | 65790.13 | 65758.35 | 65725.74 |  |  | |  | |  | 4621 | 1 - 1 | 100% |
|  | 2 | -28639.6 | 57414.22 | 57363.38 | 57311.21 | 0.999 | 0 | | 0 | | 0 | 4621 | 0.997 - 1 | 93.5%, 6.5% |
|  | 3 | -26339.1 | 52863.82 | 52793.92 | 52722.18 | 0.999 | 0 | | 0.0001 | | 0.0001 | 4621 | 0.997 - 1 | 89.5%, 6.5%, 4% |
|  | 4 | -25737.5 | 51711.25 | 51622.27 | 51530.97 | 0.978 | 0 | | 0 | | 0 | 4621 | 0.964 - 0.998 | 81.2%, 8.3%, 4%, 6.5% |
|  | 5 | -25533.8 | 51354.57 | 51246.53 | 51135.67 | 0.986 | 0 | | 0 | | 0 | 4621 | 0.972 - 0.997 | 8.5%, 81%, 1.1%, 6.6%, 2.8% |
|  | 6 | -25402.1 | 51141.65 | 51014.54 | 50884.11 | 0.878 | 0 | | 0 | | 0 | 4621 | 0.655 - 0.996 | 23.4%, 57.7%, 1.1%, 8.4%, 6.6%, 2.8% |
| 2010 |  |  |  |  |  |  |  | |  | |  |  |  |  |
|  | 1 | -48179.2 | 96446.54 | 96414.76 | 96378.47 |  |  | |  | |  | 6678 | 1 - 1 | 100% |
|  | 2 | -42192.5 | 84525.98 | 84475.13 | 84417.07 | 0.998 | 0 | | 0 | | 0 | 6678 | 0.996 - 1 | 92.3%, 7.7% |
|  | 3 | -38930 | 78053.73 | 77983.82 | 77903.99 | 0.998 | 0 | | 0 | | 0 | 6678 | 0.995 - 1 | 87.6%, 7.5%, 4.8% |
|  | 4 | -38146.4 | 76539.39 | 76450.41 | 76348.8 | 0.966 | 0 | | 0 | | 0 | 6678 | 0.951 - 0.996 | 7.5%, 4.8%, 10.1%, 77.6% |
|  | 5 | -37924.4 | 76148.21 | 76040.16 | 75916.78 | 0.975 | 0 | | 0 | | 0 | 6678 | 0.9 - 0.995 | 1.5%, 77.3%, 7.5%, 10.4%, 3.3% |
|  | 6 | -37714.5 | 75781.18 | 75654.07 | 75508.92 | 0.88 | 0 | | 0 | | 0 | 6678 | 0.7 - 0.996 | 57%, 20.6%, 10.1%, 7.5%, 1.5%, 3.3% |
| 2014 |  |  |  |  |  |  |  | |  | |  |  |  |  |
|  | 1 | -44996.3 | 90080.66 | 90048.88 | 90012.66 |  |  | |  | |  | 6634 | 1 - 1 | 100% |
|  | 2 | -39540.7 | 79222.16 | 79171.32 | 79113.36 | 0.998 | 0 | | 0 | | 0 | 6634 | 0.99 - 1 | 95.5%, 4.5% |
|  | 3 | -35647 | 71487.69 | 71417.78 | 71338.09 | 0.997 | 0 | | 0.0001 | | 0.0001 | 6634 | 0.988 - 1 | 4.5%, 3.8%, 91.8% |
|  | 4 | -22398.9 | 45044.22 | 44955.24 | 44853.82 | 0.998 | 0 | | 1 | | 1 | 6634 | 0.99 - 1 | 91.7%, 2%, 2.4%, 3.8% |
|  | 5 | -21357.7 | 43014.59 | 42906.55 | 42783.4 | 0.988 | 0 | | 1 | | 1 | 6634 | 0.981 - 0.996 | 85.5%, 6.2%, 3.8%, 2%, 2.4% |
|  | 6 | -21074.2 | 42500.32 | 42373.21 | 42228.33 | 0.883 | 0 | | 1 | | 1 | 6634 | 0.747 - 0.991 | 27.7%, 58%, 6%, 2.4%, 3.8%, 2% |
| 2018 |  |  |  |  |  |  |  | |  | |  |  |  |  |
|  | 1 | -45437.6 | 90964.41 | 90932.63 | 90895.17 |  |  | |  | |  | 7510 | 1 - 1 | 100% |
|  | 2 | -38410.3 | 76963.47 | 76912.63 | 76852.69 | 0.999 | 0 | | 0 | | 0 | 7510 | 0.993 - 1 | 3%, 97% |
|  | 3 | -35431.3 | 71058.91 | 70989 | 70906.58 | 0.999 | 0 | | 0 | | 0 | 7510 | 0.991 - 1 | 1.7%, 96.1%, 2.2% |
|  | 4 | -31712.9 | 63675.73 | 63586.75 | 63481.86 | 0.999 | 0 | | 0.0003 | | 0.0004 | 7510 | 0.99 - 1 | 1.3%, 93.6%, 3.4%, 1.7% |
|  | 5 | -29229.6 | 58762.62 | 58654.57 | 58527.2 | 0.999 | 0 | | 0.1367 | | 0.1407 | 7510 | 0.989 - 1 | 2.5%, 1.1%, 1.6%, 1.2%) 93.6% |
|  | 6 | -27988 | 56332.97 | 56205.86 | 56056.01 | 0.996 | 0 | | 0.0002 | | 0.0002 | 7510 | 0.983 - 0.999 | 3.3%, 1.2%, 90.3%, 2.5%, 1.6%, 1.1% |

*Note.* Overall = across all survey years, k = number of classes, LL = Log-Likelihood, AIC = Akaike Information Criterion, BIC = Bayesian Information Criterion, aBIC = sample size-adjusted BIC, BLRT = Bootstrapped Likelihood Ratio Test, VLMR = Vuong-Lo-Mendell-Rubin Likelihood Ratio Test, LMR = Lo-Mendell-Rubin Likelihood Ratio Test.

Table S3. Regression results of the health-risk behavior profiles: mental distress association for males and females. Health Behaviour in School-Aged Children study, Switzerland, 2002-2018.

|  | Males | | | | | | Females | |  | |  | |
| --- | --- | --- | --- | --- | --- | --- | --- | --- | --- | --- | --- | --- |
|  | Somatic symptoms | | Internalizing | | Life satisfaction | | Somatic symptoms | | Internalizing | | Life satisfaction | |
|  | β | 95% CI | β | 95%CI | β | 95%CI | β | 95%CI | β | 95%CI | β | 95%CI |
| **Reference category: low-risk** |  |  |  |  |  |  |  |  |  |  |  |  |
| 2002 |  |  |  |  |  |  |  |  |  |  |  |  |
| High alcohol use/slightly elevated substance use | .09*** | -.02, .20 | .11*** | -.00, .22 | -.07*** | -.19, .04 | .06** | -.11, .23 | .07*** | -.10, .24 | -.04 | -.20, .13 |
| Moderate substance use | .11*** | -.03, .25 | .11*** | -.03, .25 | -.07** | -.21, .08 | .10*** | -.05, .26 | .13*** | -.02, .29 | -.11*** | -.26, .04 |
| Highest risk | .13*** | -.02, .28 | **.16***** | .02, .31 | -.12*** | -.28, .03 | .15*** | -.03, .33 | **.20***** | .03, .38 | -.16*** | -.33, .02 |
| 2006 |  |  |  |  |  |  |  |  |  |  |  |  |
| High alcohol use/slightly elevated substance use | .06* | -.07, .18 | .04 | -.09, .17 | -.05* | -.18, .08 | .12*** | -.07, .31 | .09*** | -.09, .28 | -.10*** | -.28, .09 |
| Moderate substance use | .09*** | -.09, .27 | .09*** | -.10, .27 | -.10*** | -.29, .09 | .04 | -.19, .27 | .08*** | -.15, .31 | -.12*** | -.34, .11 |
| Highest risk | .08*** | -.08, .24 | .09*** | -.08, .26 | -.11*** | -.29, .06 | .12*** | -.06, .30 | .16*** | -.02, .33 | -.17*** | -.34, .00 |
| 2010 |  |  |  |  |  |  |  |  |  |  |  |  |
| High alcohol use/slightly elevated substance use | **.11***** | .01, .21 | .09*** | -.01, .19 | -.01 | -.12, .09 | .09*** | -.04, .22 | .10*** | -.04, .23 | -.07*** | -.20, .06 |
| Moderate substance use | .09*** | -.02, .21 | .09*** | -.02, .21 | -.08*** | -.20, .04 | .13*** | -.02, .27 | **.16***** | .01, .30 | **-.15***** | -.29, -.00 |
| Highest risk | .12*** | -.03, .27 | .10*** | -.05, .25 | -.08*** | -.23, .07 | .11*** | -.07, .28 | .12*** | -.06, .30 | -.14*** | -.31, .03 |
| 2014 |  |  |  |  |  |  |  |  |  |  |  |  |
| High alcohol use/slightly elevated substance use | .05** | -.11, .20 | .06*** | -.10, .22 | -.03 | -.19, .13 | .08*** | -.12, .28 | .14*** | -.06, .34 | -.09*** | -.29, .11 |
| Moderate substance use | .02*** | -.20, .24 | .05** | -.17, .27 | -.08*** | -.31, .14 | .09*** | -.17, .36 | .10*** | -.16, .36 | -.10*** | -.36, .16 |
| Highest risk | .09*** | -.11, .29 | .09*** | -.12, .29 | -.06** | -.27, .15 | .10*** | -.14, .35 | .07*** | -.17, .32 | -.07*** | -.32, .17 |
| 2018 |  |  |  |  |  |  |  |  |  |  |  |  |
| High alcohol use/slightly elevated substance use | .04** | -.12, .21 | .06*** | -.09, .22 | -.04** | -.21, .12 | .08*** | -.11, .27 | .13*** | -.06, .32 | -.09*** | -.28, .10 |
| Moderate substance use | .01 | -.24, .26 | .04* | -.21, .28 | -.00 | -.26, .25 | .04* | -.27, .34 | .08*** | -.24, .39 | -.07*** | -.38, .24 |
| Highest risk | .08*** | -.13, .29 | .09*** | -.11, .30 | -.08*** | -.30, .14 | .11*** | -.19, .41 | .08*** | -.22, .39 | -.08*** | -.38, .22 |
|  |  |  |  |  |  |  |  |  |  |  |  |  |
| **Reference category: high alcohol use/slightly elevated substance use** | | |  |  |  |  |  |  |  |  |  |  |
| 2002 |  |  |  |  |  |  |  |  |  |  |  |  |
| Moderate substance use | .04 | -.12, .21 | .03 | -.13, .19 | -.01 | -.18, .16 | .04 | -.17, .25 | .06* | -.15, .27 | -.07* | -.28, .14 |
| Highest risk | .06* | -.10, .22 | .08** | -.09, .24 | -.07** | -.24, .10 | .09*** | -.13, .32 | .14*** | -.09, .36 | -.12*** | -.34, .10 |
| 2006 |  |  |  |  |  |  |  |  |  |  |  |  |
| Moderate substance use | .05* | -.15, .26 | .06* | -.15, .27 | -.07** | -.28, .15 | -.05* | -.34, .23 | .01 | -.28, .29 | -.04 | -.32, .24 |
| Highest risk | .04 | -.15, .23 | .06* | -.13, .26 | -.08** | -.27, .12 | -.01 | -.25, .24 | .06 | -.19, .30 | -.07* | -.30, .17 |
| 2010 |  |  |  |  |  |  |  |  |  |  |  |  |
| Moderate substance use | .00 | -.14, .14 | .02 | -.12, .16 | -.07** | -.21, .08 | .05* | -.14, .23 | .07** | -.11, .26 | -.08*** | -.26, .10 |
| Highest risk | .05* | -.12, .21 | .04 | -.12, .20 | -.07*** | -.24, .10 | .04 | -.17, .25 | .05* | -.16, .26 | -.09*** | -.29, .12 |
| 2014 |  |  |  |  |  |  |  |  |  |  |  |  |
| Moderate substance use | -.02 | -.28, .25 | .01 | -.26, .27 | -.06** | -.33, .21 | .03 | -.29, .36 | -.01 | -.33, .32 | -.03 | -.35, .29 |
| Highest risk | .05* | -.20, .30 | .04 | -.21, .29 | -.04 | -.29, .22 | .04 | -.27, .35 | -.04 | -.35, .27 | -.00 | -.31, .31 |
| 2018 |  |  |  |  |  |  |  |  |  |  |  |  |
| Moderate substance use | -.02 | -.31, .28 | -.01 | -.29, .28 | .03 | -.27, .33 | -.01 | -.37, .34 | -.01 | -.36, .35 | -.02 | -.37, .34 |
| Highest risk | .04* | -.22, .31 | .04* | -.21, .30 | -.05* | -.31, .22 | .06** | -.29, .41 | -.00 | -.35, .35 | -.02 | -.37, .32 |
|  |  |  |  |  |  |  |  |  |  |  |  |  |
| **Reference category: highest risk** |  |  |  |  |  |  |  |  |  |  |  |  |
| 2002 |  |  |  |  |  |  |  |  |  |  |  |  |
| Moderate substance use | -.02 | -.20, .17 | -.05 | -.23, .13 | .06* | -.14, .25 | -.07* | -.28, .15 | -.09** | -.31, .12 | .07* | -.15, .28 |
| 2006 |  |  |  |  |  |  |  |  |  |  |  |  |
| Moderate substance use | .02 | -.21, .25 | .01 | -.23, .24 | .00 | -.24, .24 | -.05 | -.33, .23 | -.03 | -.31, .24 | .01 | -.26, .28 |
| 2010 |  |  |  |  |  |  |  |  |  |  |  |  |
| Moderate substance use | -.06* | -.23, .12 | -.03 | -.21, .14 | .02 | -.16, .20 | -.00 | -.22, .22 | .02 | -.20, .23 | .02 | -.19, .24 |
| 2014 |  |  |  |  |  |  |  |  |  |  |  |  |
| Moderate substance use | -.06** | -.35, .23 | -.03 | -.32, .26 | -.03 | -.33, .27 | -.00 | -.35, .35 | .03 | -.33, .38 | -.03 | -.38, .32 |
| 2018 |  |  |  |  |  |  |  |  |  |  |  |  |
| Moderate substance use | -.05* | -.38, .27 | -.04* | -.36, .27 | .07** | -.26, .39 | -.07** | -.50, .35 | -.00 | -.43, .42 | .01 | -.42, .43 |

*Note.* Results from multivariate models adjusted for age and family SES. Bolded coefficients indicate that the confidence interval does not cross zero.

**p*<.05; ***p*<.01; ****p*<.001;

Table S4. Results from the health-risk behavior*sex interaction predicting mental distress. Health Behaviour in School-Aged Children study, Switzerland, 2002-2018.

|  | Profile*sex interaction | | | | | |
| --- | --- | --- | --- | --- | --- | --- |
|  | Somatic symptoms | | Internalizing | | Life satisfaction | |
|  | β | 95% CI | β | 95%CI | β | 95%CI |
| **Reference category: low-risk** |  |  |  |  |  |  |
| 2002 |  |  |  |  |  |  |
| High alcohol use/slightly elevated substance use | .05 | -.14, .24 | .05 | -.14, .24 | -.00 | -.20, .19 |
| Moderate substance use | .05 | -.16, .25 | .11* | -.10, .31 | -.10* | -.30-.11 |
| Highest risk | .13** | -.08, .35 | .19*** | -.02, .41 | -.12** | -.34, .10 |
| 2006 |  |  |  |  |  |  |
| High alcohol use/slightly elevated substance use | .19*** | -.03, .41 | .16*** | -.06, .38 | -.12** | -.34, .09 |
| Moderate substance use | -.02 | -.31, .27 | .05 | -.24, .34 | -.08 | -.37, .21 |
| Highest risk | .13** | -.10, .37 | .17*** | -.07, .40 | -.12** | -.36, .11 |
| 2010 |  |  |  |  |  |  |
| High alcohol use/slightly elevated substance use | .07 | -.09, .23 | .07 | -.09, .23 | -.10*** | -.26, .06 |
| Moderate substance use | .14*** | -.04, .32 | .16*** | -.02, .34 | -.14** | -.32, .04 |
| Highest risk | .07 | -.15, .29 | .08* | -.14, .31 | -.13*** | -.35, .10 |
| 2014 |  |  |  |  |  |  |
| High alcohol use/slightly elevated substance use | .09* | -.16, .34 | .17*** | -.08, .43 | -.12*** | -.38, .13 |
| Moderate substance use | .15*** | -.19, .49 | .11** | -.22, .45 | -.06 | -.40, .28 |
| Highest risk | .08* | -.23, .39 | .04 | -.28, .35 | -.06 | -.38, .26 |
| 2018 |  |  |  |  |  |  |
| High alcohol use/slightly elevated substance use | .10** | -.14, .35 | .16*** | -.08, .41 | -.10** | -.35, .15 |
| Moderate substance use | .07* | -.32, .46 | .11** | -.28, .50 | -.13*** | -.53, .26 |
| Highest risk | .13*** | -.23, .49 | .06 | -.30, .42 | -.06 | -.42, .30 |
|  |  |  |  |  |  |  |
| **Reference category: high alcohol use/slightly elevated substance use** |  |  |  |  |  |  |
| 2002 |  |  |  |  |  |  |
| Moderate substance use | -.00 | -.27, .26 | .05 | -.21, .32 | -.10 | -.36, .17 |
| Highest risk | .09 | -.18, .37 | .15* | -.13, .42 | -.12* | -.39, .16 |
| 2006 |  |  |  |  |  |  |
| Moderate substance use | -.16** | -.51, .19 | -.07 | -.42, .28 | .02 | -.33, .37 |
| Highest risk | -.05 | -.36, .25 | .01 | -.30, .32 | .00 | -.30, .31 |
| 2010 |  |  |  |  |  |  |
| Moderate substance use | .08 | -.15, .30 | .10* | -.13, .33 | -.05 | -.28, .18 |
| Highest risk | .02 | -.24, .28 | .03 | -.23, .30 | -.05 | -.32, .21 |
| 2014 |  |  |  |  |  |  |
| Moderate substance use | .08 | -.33, .50 | -.02 | -.43, .40 | .03 | -.39, .45 |
| Highest risk | .00 | -.39, .40 | -.10* | -.50, .29 | .04 | -.36, .44 |
| 2018 |  |  |  |  |  |  |
| Moderate substance use | .01 | -.45, .47 | .00 | -.45, .46 | -.07 | -.54, .39 |
| Highest risk | .06 | -.37, .49 | -.05 | -.48, .38 | .01 | -.42, .44 |
|  |  |  |  |  |  |  |
| **Reference category: highest risk** |  |  |  |  |  |  |
| 2002 |  |  |  |  |  |  |
| Moderate substance use | -.10 | -.39, .18 | -.11 | -.39, .17 | .03 | -.25, .32 |
| 2006 |  |  |  |  |  |  |
| Moderate substance use | -.12* | -.48, .24 | -.08 | -.44, .29 | .02 | -.35, .38 |
| 2010 |  |  |  |  |  |  |
| Moderate substance use | .05 | -.22, .33 | .06 | -.22, .34 | .01 | -.26, .29 |
| 2014 |  |  |  |  |  |  |
| Moderate substance use | .08 | -.38, .53 | .08 | -.38, .54 | -.01 | -.47, .45 |
| 2018 |  |  |  |  |  |  |
| Moderate substance use | -.05 | -.58, .48 | .05 | -.47, .58 | -.08 | -.61, .45 |

*Note.* Results from multivariate models adjusted for age and family SES.

**p*<.05; ***p*<.01; ****p*<.001

Table S5. Associations of sociodemographic characteristics with health-risk behavior profiles estimated across all waves. Health Behaviour in School-Aged Children study, Switzerland, 2002-2018.

|  | Slightly elevated substance use vs. Low-risk | | Moderate substance use vs. Low-risk | | Highest risk vs. Low-risk | | Moderate substance use vs. Slightly elevated substance use | | Highest risk vs. Slightly elevated substance use | | Moderate substance use vs. Highest risk | |
| --- | --- | --- | --- | --- | --- | --- | --- | --- | --- | --- | --- | --- |
|  | OR | 95%CI | OR | 95%CI | OR | 95%CI | OR | 95%CI | OR | 95%CI | OR | 95%CI |
| 2002 |  |  |  |  |  |  |  |  |  |  |  |  |
| Sex | 0.93 | 0.70-1.23 | 1.16 | 0.84-1.59 | 0.81 | 0.63-1.02 | 1.25 | 0.83-1.87 | 0.87 | 0.61-1.23 | 1.43 | 0.99, 2.09 |
| SES | 0.89 | 0.78-1.03 | 1.09 | 0.93-1.28 | 0.89 | 0.79-1.01 | 1.22 | 1.00-1.51 | 1.00 | 0.84-1.19 | **1.22*** | 1.01, 1.48 |
| Age | **1.76***** | 1.51-2.04 | **2.92***** | 2.38-3.57 | **4.05***** | 3.41-4.83 | **1.66***** | 1.30-2.12 | **2.31***** | 1.85-2.88 | **0.72*** | 0.55, 0.93 |
| 2006 |  |  |  |  |  |  |  |  |  |  |  |  |
| Sex | **0.68*** | 0.50-0.92 | 1.22 | 0.83-1.79 | 0.79 | 0.57-1.08 | **1.79*** | 1.12, 2.88 | 1.15 | 0.76, 1.75 | 1.55 | 0.96, 2.50 |
| SES | 0.88 | 0.76-1.02 | 0.92 | 0.76-1.11 | **0.85*** | 0.73-0.99 | 1.05 | 0.83, 1.32 | 0.97 | 0.79, 1.18 | 1.09 | 0.86, 1.38 |
| Age | **2.01***** | 1.70-2.38 | **2.45***** | 1.95-3.08 | **6.04***** | 4.51-8.10 | 1.22 | 0.92, 1.61 | **3.00***** | 2.15, 4.19 | **0.41***** | 0.28, 0.58 |
| 2010 |  |  |  |  |  |  |  |  |  |  |  |  |
| Sex | **0.61***** | 0.48-0.77 | 0.78 | 0.58-1.06 | **0.73**** | 0.57-0.92 | 1.29 | 0.89, 1.87 | 1.19 | 0.87, 1.65 | 1.08 | 0.75, 1.56 |
| SES | 1.04 | 0.93-1.17 | 1.08 | 0.93-1.25 | 0.90 | 0.80-1.01 | 1.03 | 0.86, 1.24 | 0.86 | 0.74, 1.01 | 1.20 | 1.00-1.43 |
| Age | **2.19***** | 1.90-2.51 | **3.07***** | 2.50-3.76 | **4.61***** | 3.80-5.59 | **1.40**** | 1.10, 1.79 | **2.11***** | 1.67, 2.66 | **0.67**** | 0.51, 0.88 |
| 2014 |  |  |  |  |  |  |  |  |  |  |  |  |
| Sex | **0.73*** | 0.56-0.95 | 0.82 | 0.58-1.17 | 0.81 | 0.58-1.12 | 1.13 | 0.73, 1.73 | 1.10 | 0.73, 1.66 | 1.02 | 0.64, 1.63 |
| SES | **1.22**** | 1.06-1.41 | 1.00 | 0.84-1.20 | 0.92 | 0.77-1.08 | 0.82 | 0.65, 1.03 | **0.75**** | 0.60, 0.93 | 1.10 | 0.86, 1.40 |
| Age | **1.93***** | 1.67-2.23 | **2.62***** | 2.09-3.29 | **5.46***** | 4.05-7.36 | **1.36*** | 1.04, 1.77 | **2.83***** | 2.04, 3.93 | **0.48***** | 0.33, 0.70 |
| 2018 |  |  |  |  |  |  |  |  |  |  |  |  |
| Sex | 0.98 | 0.76-1.27 | 0.86 | 0.57-1.29 | **0.65*** | 0.44-0.95 | 0.88 | 0.54, 1.41 | 0.66 | 0.42, 1.04 | 1.32 | 0.77, 2.29 |
| SES | 1.09 | 0.95-1.25 | 1.07 | 0.87-1.31 | 0.87 | 0.73-1.03 | 0.98 | 0.77, 1.25 | **0.79*** | 0.64, 0.98 | 1.23 | 0.95, 1.60 |
| Age | **2.93***** | 2.46-3.48 | **6.28***** | 4.22-9.33 | **6.01***** | 4.22-8.56 | **2.14***** | 1.40, 3.29 | **2.05***** | 1.39, 3.03 | 1.04 | 0.62, 1.76 |

*Note.* OR=odds ratio, CI=confidence interval.

Table S6. Regression results of the health-risk behavior profiles-mental distress association with health-risk behavior profiles estimated across all waves. Health Behaviour in School-Aged Children study, Switzerland, 2002-2018.

|  | Bivariate | | | | | | Multivariate | | | | | |
| --- | --- | --- | --- | --- | --- | --- | --- | --- | --- | --- | --- | --- |
|  | Somatic symptoms | | Internalizing | | Life satisfaction | | Somatic symptoms | | Internalizing | | Life satisfaction | |
|  | β | 95% CI | β | 95%CI | β | 95%CI | β | 95%CI | β | 95%CI | β | 95%CI |
| **Reference category: low-risk** |  |  |  |  |  |  |  |  |  |  |  |  |
| 2002 |  |  |  |  |  |  |  |  |  |  |  |  |
| Slightly elevated substance use | .07*** | -.07, .20 | .08*** | -.06, .21 | -.07*** | -.20, .07 | .07*** | -.07, .20 | .08*** | -.06, .21 | -.06*** | -.20, .07 |
| Moderate substance use | .08*** | .,07,.23 | .09******* | -.06, .24 | -.06*** | -.21, .09 | .07******* | -.08, .22 | .08******* | -.07, .23 | -.05*** | -.21, .10 |
| Highest risk | **.12***** | .02, .24 | **.16***** | .05, .27 | **-.15***** | -.26, -.04 | **.12***** | .01, .23 | **.16***** | .05, .27 | **-.13***** | -.24, -.02 |
| 2006 |  |  |  |  |  |  |  |  |  |  |  |  |
| Slightly elevated substance use | .05*** | -.09, .21 | .08*** | -.07, .23 | -.12*** | -.26, .03 | .05*** | -.09, .20 | .08*** | -.07, .22 | -.10*** | -.25, .04 |
| Moderate substance use | .06*** | -.13, .24 | .09*** | -.10, .27 | -.10*** | -.28, .09 | .04** | -.14, .23 | .08*** | -.11, .26 | -.08*** | -.27, .11 |
| Highest risk | .10*** | -.05, .25 | .10******* | -.05, .25 | -.14******* | -.29, .01 | .08*** | -.07, .23 | .09******* | -.06, .24 | -.11******* | -.26, .04 |
| 2010 |  |  |  |  |  |  |  |  |  |  |  |  |
| Slightly elevated substance use | .07*** | -.05, .18 | .08*** | -.03, .20 | -.09*** | -.20, .02 | .06*** | -.05, .18 | .09*** | -.03, .20 | -.08*** | -.19, .03 |
| Moderate substance use | .08*** | -.07, .22 | .07*** | -.08, .21 | -.08******* | -.23, .06 | .07*** | -.08, .21 | .07*** | -.08, .21 | -.07******* | -.21, .07 |
| Highest risk | .11*** | -.00, .22 | .10*** | -.01, .21 | **-.13***** | -.24, -.02 | .09*** | -.02, .21 | .09*** | -.02, .21 | -.10*** | -.21, .01 |
| 2014 |  |  |  |  |  |  |  |  |  |  |  |  |
| Slightly elevated substance use | .07*** | -.06, .19 | .09*** | -.03, .22 | -.06*** | -.19, .07 | .06*** | -.06, .19 | .10*** | -.03, .22 | -.06*** | -.18, .07 |
| Moderate substance use | .06*** | -.11, .24 | .07*** | -.10, .24 | -.10*** | -.27, .08 | .06*** | -.12, .22 | .07*** | -.10, .24 | -.09*** | -.26, .09 |
| Highest risk | .11*** | -.05, .26 | .08*** | -.07, .24 | -.08*** | -.24, .08 | .09*** | -.06, .25 | .08*** | -.08, .24 | -.07*** | -.23, .09 |
| 2018 |  |  |  |  |  |  |  |  |  |  |  |  |
| Slightly elevated substance use | .08*** | -.05, .20 | .11*** | -.02, .23 | -.09*** | -.21, .04 | .06*** | -.06, .19 | .10*** | -.03, .22 | -.05*** | -.18, .08 |
| Moderate substance use | .04** | -.16, .24 | .06*** | -.14, .26 | -.05*** | -.25, .15 | .02* | -.17, .22 | .05*** | -.14, .25 | -.05*** | -.25, .16 |
| Highest risk | .10*** | -.08, .27 | .08*** | -.10, .26 | -.09*** | -.27, .09 | .09*** | -.09, .27 | .08*** | -.10, .26 | -.10*** | -.29, .08 |
|  |  |  |  |  |  |  |  |  |  |  |  |  |
| **Reference category: slightly elevated substance use** | | |  |  |  |  |  |  |  |  |  |  |
| 2002 |  |  |  |  |  |  |  |  |  |  |  |  |
| Moderate substance use | .02 | -.17, .22 | .02 | -.17, .22 | -.00 | -.20, .19 | .01 | -.18, .21 | .01 | -.18, .21 | .00 | -.20, .20 |
| Highest risk | .05* | -.12, .21 | .07** | -.10, .24 | -.07** | -.23, .10 | .04 | -.13, .21 | .07** | -.10, .23 | -.05* | -.22, .11 |
| 2006 |  |  |  |  |  |  |  |  |  |  |  |  |
| Moderate substance use | .01 | -.22, .24 | .02 | -.21, .26 | -.01 | -.24, .23 | -.00 | -.23, .23 | .01 | -.22, .25 | -.00 | -.23, .23 |
| Highest risk | .04* | -.16, .25 | .02 | -.18, .23 | -.02 | -.23, .18 | .03 | -.17, .23 | .02 | -.19, .22 | -.01 | -.21, .19 |
| 2010 |  |  |  |  |  |  |  |  |  |  |  |  |
| Moderate substance use | .03 | -.15, .21 | .01 | -.18, .19 | -.01 | -.20, .17 | .01 | -.16, .19 | -.00 | -.18, .18 | -.01 | -.19, .17 |
| Highest risk | .04* | -.11, .20 | .02 | -.14, .17 | -.04* | -.20, .11 | .03 | -.12, .18 | .00 | -.15, .16 | -.02 | -.18, .13 |
| 2014 |  |  |  |  |  |  |  |  |  |  |  |  |
| Moderate substance use | .01 | -.20, .22 | .00 | -.21, .21 | -.05*** | -.26, .16 | .01 | -.20, .22 | -.00 | -.21, .21 | -.05** | -.25, .16 |
| Highest risk | .05*** | -.14, .25 | .01 | -.19, .21 | -.03* | -.23, .17 | .04** | -.15, .24 | .00 | -.19, .20 | -.02 | -.22, .18 |
| 2018 |  |  |  |  |  |  |  |  |  |  |  |  |
| Moderate substance use | -.01 | -.24, .22 | -.01 | -.24, .22 | .00 | -.23, .24 | -.02 | -.24, .21 | -.01 | -.24, .22 | .01 | -.23, .24 |
| Highest risk | .04 | -.17, .26 | .01 | -.21, .22 | -.03* | -.25, .18 | .04** | -.17, .26 | .01 | -.20, .22 | -.03* | -.24, .18 |
|  |  |  |  |  |  |  |  |  |  |  |  |  |
| **Reference category: highest risk** |  |  |  |  |  |  |  |  |  |  |  |  |
| 2002 |  |  |  |  |  |  |  |  |  |  |  |  |
| Moderate substance use | -.01 | -.19, .17 | -.03 | -.21, .15 | 0.04* | -0.14, .22 | -.02 | -.20, .16 | -.04* | -.21, .14 | .04* | -.14, .22 |
| 2006 |  |  |  |  |  |  |  |  |  |  |  |  |
| Moderate substance use | -.02 | -.26, .21 | .00 | -.23, .24 | .01 | -.22, .25 | -.02 | -.26, .21 | .00 | -.23, .23 | .01 | -.23, .24 |
| 2010 |  |  |  |  |  |  |  |  |  |  |  |  |
| Moderate substance use | -.01 | -.19, .17 | -.01 | -.19, .17 | .02 | -.16, .20 | -.01 | -.18, .17 | -.01 | -.18, .17 | .02 | -.16, .20 |
| 2014 |  |  |  |  |  |  |  |  |  |  |  |  |
| Moderate substance use | -.03* | -.26, .20 | -.00 | -.23, .22 | -.02 | -.25, .21 | -.03 | -.26, .20 | -.00 | -.23, .23 | -.03 | -.26, .20 |
| 2018 |  |  |  |  |  |  |  |  |  |  |  |  |
| Moderate substance use | -.05** | -.31, .21 | -.01 | -.28, .25 | .03 | -.24, .30 | -.06*** | -.32, .21 | -.02 | -.28, .24 | .03* | -.23, .30 |

*Note.* Multivariate models adjusted for sex, age, and family SES. Coefficients are bolded when the confidence interval does not cross zero.

**p*<0.05; ***p*<0.01; ****p*<0.001
